# Wearable Electrocardiogram Quality Assessment Using Wavelet Scattering and LSTM

**DOI:** 10.3389/fphys.2022.905447

**Published:** 2022-06-30

**Authors:** Feifei Liu, Shengxiang Xia, Shoushui Wei, Lei Chen, Yonglian Ren, Xiaofei Ren, Zheng Xu, Sen Ai, Chengyu Liu

**Affiliations:** ^1^ School of Science, Shandong Jianzhu University, Jinan, China; ^2^ School of Control Science and Engineering, Shandong University, Jinan, China; ^3^ School of Science and Technology, Shandong University of Traditional Chinese Medicine, Jinan, China; ^4^ School of Information and Electrical Engineering, Shandong Jianzhu University, Jinan, China; ^5^ School of Instrument Science and Engineering, Southeast University, Nanjing, China

**Keywords:** dynamic electrocardiogram, signal-quality assessment, wavelet scattering, signal-quality index, long short-term memory network

## Abstract

As the fast development of wearable devices and Internet of things technologies, real-time monitoring of ECG signals is quite critical for cardiovascular diseases. However, dynamic ECG signals recorded in free-living conditions suffered from extremely serious noise pollution. Presently, most algorithms for ECG signal evaluation were designed to divide signals into acceptable and unacceptable. Such classifications were not enough for real-time cardiovascular disease monitoring. In the study, a wearable ECG quality database with 50,085 recordings was built, including A/B/C (or high quality/medium quality/low quality) three quality grades (A: high quality signals can be used for CVD detection; B: slight contaminated signals can be used for heart rate extracting; C: heavily polluted signals need to be abandoned). A new SQA classification method based on a three-layer wavelet scattering network and transfer learning LSTM was proposed in this study, which can extract more systematic and comprehensive characteristics by analyzing the signals thoroughly and deeply. Experimental results (***mACC*** = 98.56%, ***mF***_**1**_ = 98.55%, ***Se***_A_ = 97.90%, ***Se***_B_ = 98.16%, ***Se***_C_ = 99.60%, + ***P***_A_ = 98.52%, + ***P***_B_ = 97.60%, + ***P***_C_ = 99.54%, ***F***_1A_ = 98.20%, ***F***_1B_ = 97.90%, ***F***_1C_ = 99.60%) and real data validations proved that this proposed method showed the high accuracy, robustness, and computationally efficiency. It has the ability to evaluate the long-term dynamic ECG signal quality. It is advantageous to promoting cardiovascular disease monitoring by removing contaminating signals and selecting high-quality signal segments for further analysis.

## Introduction

Cardiovascular diseases (CVDs) are the most common non-communicable diseases globally, responsible for an estimated 17.8 million deaths in 2017, accounting for 31% of all global deaths, of which more than three quarters were in low income and middle-income countries (Liu et al., 2018; Roth et al., 2018). Therefore, early continuous monitoring and prevention for CVDs are very urgent. The recent commercial availability of wearable devices and Internet of things (IoT) technologies with cardiovascular disease detection capabilities has revolutionized the diagnosis and management of these common medical issues, as it has placed the power of arrhythmia detection into the hands of the patient (Liu et al., 2019b). However, the dynamic long-term ECG signals suffer from extremely serious noise pollution due to the dynamic long-term unsupervised free-living monitoring environment (Huerta et al., 2019). A recent study of 100 patients undergoing cardioversion for atrial fibrillation showed that 34% of wearable devices’ ECG recordings were categorized as “unclassified” by the device algorithm due to unclear reasons or baseline artifacts and low amplitude recordings (Kaptoge et al., 2019). Poor electrocardiographic signal quality can result in misinterpretation and inappropriate results, hazard the correct diagnosis information (Andrea et al., 2018), increases the risk of false alerts (Liu et al., 2011), which may lead to unnecessary medical referrals and testing (Ip, 2019), and increase the workload of physicians (Zhao and Zhang, 2018). Consequently, it is quite urgent to evaluate the quality of wearable dynamic ECG signals, to eliminate signals with serious noise pollution, to distinguish between clean signals that can be used for disease diagnosis and mildly contaminated signals that can only be used for heart rate extraction, which can effectively reduce false alarm and avoid interference with CVD diagnosis (Xu et al., 2021).

The quality evaluation of wearable dynamic ECG signals has aroused the researchers’ extensive attention (Satija et al., 2018; Liu et al., 2019a; Huerta et al., 2019; Liu F. et al., 2020). As early as in 2011, the PhysioNet Cardiology Challenge addressed the issue of developing an efficient algorithm being able to run in real-time on a mobile phone, which can be able to indicate within a few seconds, while the patient is still present, if the ECG is of adequate quality for interpretation, or if another recording should be made (Silva et al., 2011). From then on, many wearable ECG signal-quality assessment (SQA) methods have been developed, and a variety of signal-quality indexes (SQI) have been explored based on the extraction of statistical, morphological, nonlinear, or time-frequency domain features etc. from the signals (Smital et al., 2020). For instance, Li et al. (2008) proposed a bSQI index based on the principle that different R-wave detectors should be nearly the same for clean ECG signals, while they should have different results for ECG signals polluted by noises, and got a good grade in the 2011 PhysioNet/CinC Challenge (Clifford and Moody, 2012). Based on this index, Liu et al. (2018) proposed the generalized bSQI index, generalized the two QRS detector–based bSQI to multiple QRS detector–based bSQI, and mainly studied the effects of type and number of R wave detectors on signal-quality assessment performances. Smital et al. (2020) proposed continuous signal-to-noise ratio curve using the time-frequency domain approach, including the Wavelet Wiener Filtering method and short-time Fourier transform frequency approach, to estimate real-time quality assessment of long-term ECG signals recorded by wearables in free-living conditions. He et al. (2020) proposed a fuzzy comprehensive evaluation algorithm based on characteristics of ECG waveform and each band, to comprehensively evaluate the quality of ECG signals.

However, existing SQA methods highly demand robust methods for accurate and reliable detection and measurement of morphological and RR-interval features from noise-free and noisy ECG signals. Although the ECG morphology feature–based methods have shown promising results in noise-free ECG recordings, accuracy and robustness of QRS complex detection and waveform delineation methods are significantly degraded in the presence of severe muscle artifacts and other external noise (Satija et al., 2017). Also, most SQA methods graded the dynamic ECG signal quality into two groups: acceptable versus unacceptable (or good versus bad). In fact, in some wearable ECG signals only R wave could be detected, other waves such as P or ST were drowned out by the noise (Xu et al., 2021). These signals cannot be used for some CVD detection, but they also cannot be abandoned as heart rate information can be obtained. Therefore, these signals could not be simply divided into acceptable or unacceptable. In this study, a wearable ECG quality database with 50,085 recordings was built, which included A/B/C (or high quality/medium quality/low quality) three quality grades (A: high-quality signals can be used for CVD detection; B: slightly contaminated signals can be used for heart rate extracting; C: heavily polluted signals need to be abandoned). The research has revealed that traditional indexes merely based on morphological, nonlinear, or time-frequency domain features did not perform well on this database, as class B signals were easily confused with class A signals. It is essential to extract more systematic and comprehensive characteristics by analyzing the signals thoroughly and deeply.

The wavelet scattering algorithm, proposed by Mallat (2012), Bruna and Mallat (2013), and Anden and Mallat (2014) using the deep convolutional network architecture, iterated over wavelet convolution, nonlinear modulus, and averaging operators to compute higher-order scattering coefficients, which can build the translation invariant, stable and informative signal representation. The wavelet transform method provided stability under the action of small diffeomorphism, while the nonlinear operation and the integration over time give translation invariance (Tang et al., 2015). Cascading wavelet transforms allowed the recovery of high frequencies lost when averaging the absolute values of coefficients of previous wavelet transforms (Destouet et al., 2021). These preprocessing methods provided an in-depth analysis of signals. First-order scattering coefficients characterize persistent phenomena such as tendency and envelope, while second-order scattering coefficients characterize transient phenomena such as shock signals and amplitude modulation (Anden and Mallat, 2014). The wavelet scattering method has been wildly used for acoustic scene classification (Li et al., 2019), speech recognition (Fousek et al., 2015; Joy et al., 2020), and heart sound classification (Mei et al., 2021), which yielded efficient representations for audio processing. However, wavelet scattering currently was seldom used in ECG analysis and application. Sepúlveda et al. (2021)extracted features of the signal at different time scales using the wavelet scattering algorithm for emotion recognition. Also, Liu Z. et al. (2020) employed wavelet scattering transform for ECG beat classification.

In this study, in order to address the classification issue of A/B/C three quality levels wearable ECG signals, a new SQA classification model was proposed based on a three-layer wavelet scattering network and transfer learning long short-term memory (LSTM) method. As the result shows, it performed very well on the quality assessment of wearable dynamic ECGs.

## Materials and Methods

**FIGURE 1:**
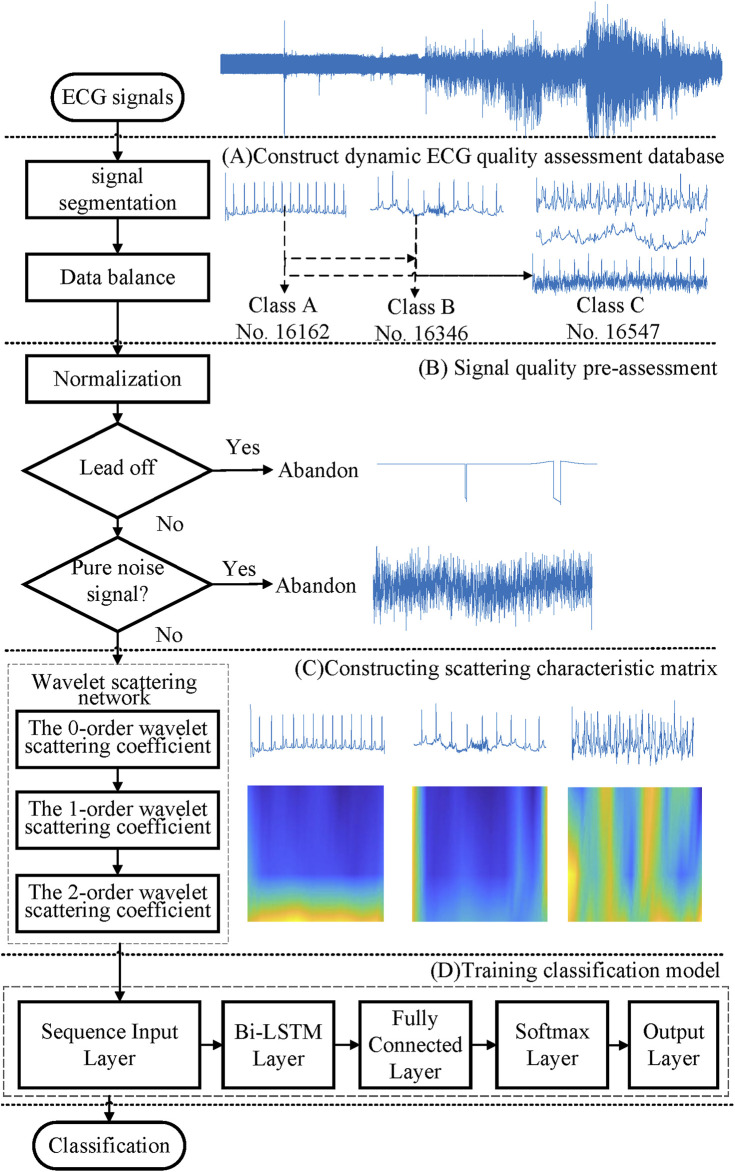
Flowchart of the proposed method.

Figure 1 displays the flowchart of the proposed method. It first established a wearable ECG quality database with 50,085 recordings from two public databases. Then, a quality pre-assessment was established, to delete the lead-fall signals and pure noise, and to avoid the adverse impact of invalid samples on the training models. Also then, the scattering characteristic matrix was extracted by applying a three-layer wavelet scattering network. Finally, a bi-directional long short-term memory (Bi-LSTM) network was employed to train the classification model.

### Database

A total of 50,085 recordings of wearable ECGs were used in this study, which were from the Brno University of Technology ECG Quality Database (BUTQDB) (Nemcova et al., 2020) and the 2011 PhysioNet/CinC Challenge (Goldberger et al., 2000; Silva et al., 2011). In the Brno University of Technology ECG Quality Database, the data comprise 18 long-term recordings of single-lead ECGs, collected from 15 subjects (9 females, six males) aged between 21 and 83 years. The signals are longer than 24 h which were detected using the Bittium Faros 180 device (mobile ECG recorder) under free-living conditions. All patients on the datasets did not have any diagnostics. The database contains signal-quality labels for partly data provided by three ECG experts, as well as the consensus of these experts, who grouped the signals into three quality classes.

Class A (high quality): all significant waveforms (P\QRS\ST\T waves) are clearly visible and the onsets and offsets of these waveforms can be detected reliably. The recording with no obvious noise can be used for the diagnosis of cardiovascular disease.

Class B (medium quality): the noise level is increased and significant points in the ECG are unclear (for example, PR interval and/or QRS duration cannot be measured reliably), but QRS complexes are clearly visible and the signal enables reliable QRS detection. Heart rate can be measured correctly.

Class C (low quality): QRS complexes cannot be detected reliably and the signal is unsuitable for any analysis. Heart rate cannot be measured correctly. These signals will interfere with the diagnosis of the cardiovascular disease and need to be removed.

In this study, the annotated recordings and segments have been divided into many fragments of unequal length based on the signal-quality labels provided by ECG experts. Each fragment has an independent label. Also, we segmented the annotated fragments into 10-s fragments with no overlap. Also, a sample of 10-s is the input data to the classification model. The number of class A is 11,708, class B is 7,860, and class C is only 657. It was obvious that data distribution was unbalanced. As we know, imbalanced classes will greatly reduce the generalization ability of the classification model (Clifford et al., 2012). Balancing the database classes can overcome this problem. In this study, we balanced the dataset by expanding the class C data using two ways: one is importing same class data from other databases, and the other is adding noise to clean data.

A total of 1,000 recordings of standard 12-lead ECGs were provided by the 2011 PhysioNet/CinC Challenge (Silva et al., 2011). In 1,000 12-lead ECGs, 773 were labeled as “acceptable,” 225 were “unacceptable,” and two were “intermediate.” Each signal had a length of 10 s. All patients on the datasets did not have any diagnostics. In Liu et al. (2018), every single channel of ECGs had been scored and re-labeled by five researchers, and a total of 9,941 acceptable and a total of 2,059 unacceptable 10-s ECG segments were obtained. In this study, based on the scores in the Liu et al. (2018) and Liu et al. (2019b), we annotated all the leads (10 s segments) individually. For every single channel of ECGs, five scores $S_{i},i=1\cdots 5$ were given by five researchers as presented in Table 1. Also, the average score $\bar{S}\left(\bar{S}=\frac{1}{5}\sum_{i=1}^{5}S_{i}\right)$ was used as a threshold. The signals were re-labeled as “class A” if it was higher than 0.75, as “class B” if it was higher than 0.25 and lower than 0.75. Otherwise, the signal was labeled as “class C”. We obtained a total of 4,455 “class A,” a total of 5,486 “class B,” and a total of 2,059 “class C” 10-s ECG segments.

**TABLE 1:** Five signal quality scores for the 10-s ECG segments.

Score	Description for signal quality scoring
1	ECGs have a clear QRS complex and T wave. Baseline wander does not influence the identification for QRS.
0.75	Transient high amplitude impulse exists, but not more than three episodes. The majority of QRS complexes can be visually clearly identified.
0.5	Both large baseline wander and transient high amplitude impulse exist. It is challenging to visually clearly identify the QRS complexes in a 2–3 s time window.
0.25	More serious lager noises exist, such as strong Gaussian noise and signal saturation and others. In these noise episodes, it is impossible to identify the QRS complex. But at least 4–5 s continuous identifiable heart beats are visible.
0	Strong noises occupy in the more than 5 s episode. It is very hard to identify the heart beat for the most signal.

If all signals from these two databases were used together simply, the number of class A would be 16,163, class B would be 13,346, and class C would be only 2,716. It was obvious that data distribution was also extremely unbalanced. In this way, class B 7860 signals from the Brno University of Technology ECG Quality Database were employed to expand the class C data by adding noise from the PhysioNet noise stress test database (NSTDB) (Moody et al., 1984). Also, 10,000 recordings chosen randomly form class A were also used to expand the class B database (3,000) and class C database (7,000) by adding noise from NSTDB, for class B; the signal-to-noise ratio (SNR) was equal to 10db, for class C was -10bd. In the NSTDB database, three types of noise were exiting, record *bw* contains baseline wander noise, record *em* contains electrode motion artifact with a significant amount of baseline wander and muscle noise as well, and record *ma* contains mainly muscle noise (Clifford et al., 2012). Because the baseline wander (*bw*) has little effect on signal quality, Gaussian noises were added to this type of noise to generate new noisy records *gbw*. Table 2 shows the details of dynamic ECG quality assessment database composition.

**TABLE 2:** Dynamic ECG quality assessment database composition.

Quality class	# Record		Source	Sampling frequency (Hz)	Record length (second)
A	16,163	11,708	BUTQDB	1,000	10
		4,455	2011 PhysioNet/CinC	500	10
B	16,346	7,860	BUTQDB	1,000	10
		5,486	2011 PhysioNet/CinC	500	10
		3,000	Class A signal set randomly + NSTDB	—	10
C	17,576	657	BUTQDB	1,000	10
		2,059	2011 PhysioNet/CinC	500	10
		7,860	Class B signal form BUTQDB + NSTDB	—	10
		7,000	Class A signal set randomly + NSTDB	—	10
Total	50,085	—	250	10	

### Signal-Quality Pre-assessment

**FIGURE 2:**
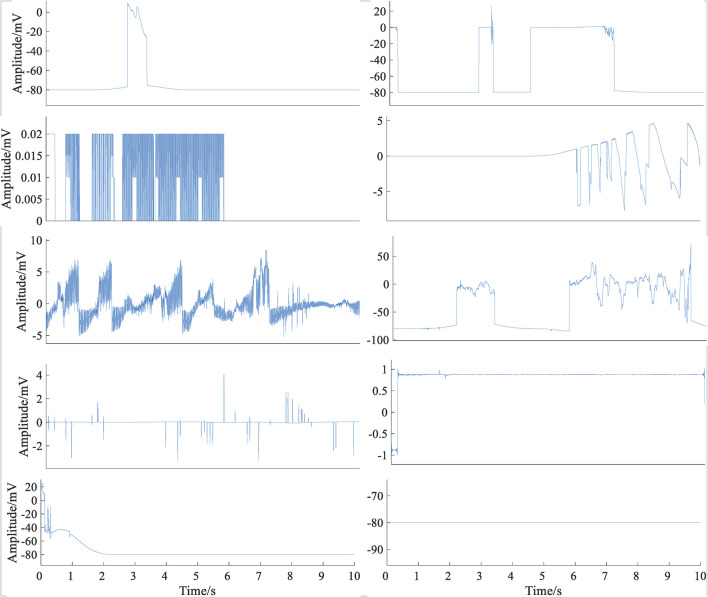
Typical cases of lead-fall signals.

Signals from different databases need to be preprocessed. First, each ECG signal was down-sampled to 250 Hz. Then, the min–max standardization method was used to map the original ECG signal data to [0–1]. Lead fall is very common in wearable dynamic ECG signals. Lead fall detection was an important way to decrease data storage costs and computing overhead for wearable devices. Figure 2 shows several typical cases of lead-fall signals. In this study, if one ECG signal was present with a constant voltage of more than 80% of the recording, it was defined as lead falling. Occasionally, detached electrodes were adhered to clothing and received Gaussian noise signals. If the signal was pure noise, it was needed to be eliminated. Based on the spectrum range of ECG signal, that is, 0–40 Hz, if the ratio of power spectrum energy of the signal in the range of 0–40 Hz to the total energy is less than 30%, it indicates that the main component of the signal is not the ECG signal but the noise signal, which can be directly discarded. By pre-assessment, 1,029 lead off and pure noise signals were eliminated, which belong to the class C. The calculation formula is as follows:

$$MPSQI=\frac{\int_{0Hz}^{40Hz}p\left(f\right)df}{\int_{0Hz}^{500Hz}p\left(f\right)df}$$ (1)

PNSQI={1pure noiseif  MPSQI<30%0 otherwise. (2)

### Wavelet Scattering Analysis

The wavelet scattering network has the characteristics of translation invariance, deformation stability, and high frequency preservation (Bruna and Mallat, 2013), and it is very sensitive to the deformation of wearable dynamic ECG signals. In this study, the scale function $\phi_{I}$ and Morlet wavelet function $\psi_{\lambda }$ were employed to construct a three-layer wavelet scattering network. Through this network, ECG signals generate scattering coefficients of order 0, 1, and 2, which can cover the whole frequency domain of the signal. The network constructing steps are as follows:

1) ECG signal X(t) was convoluted with the scale function $\phi_{I}$ to obtain the 0-order wavelet scattering coefficient $S_{0}$ .

$$S_{0}X\left(t\right)=X\left(t\right)*\phi_{I}$$ (3)

2) ECG signal X(t) were convoluted with the first-order wavelet functions, $\psi_{\lambda_{1,i}}$ , and the first-order scattering propagation operators $U_{\lambda_{1,i}}$ were generated by nonlinear modulus operation.

$$U_{\lambda_{1,i}}=\left|X\left(t\right)*\psi_{\lambda_{1,i}}\right|,\;\;\;\;i=1\cdots n.$$ (4)

3) The first-order wavelet scattering coefficients $S_{1,i}$ are obtained by the convolution of propagators $U_{\lambda_{1,i}}$ and scaling function $\phi_{I}$ .

$$S_{1,i}X\left(t\right)=\left|X\left(t\right)*\psi_{\lambda_{1,i}}\right|*\phi_{I},\;\;\;\;i=1\cdots n.$$ (5)

4) The first-order scattering propagator $U_{\lambda_{1,i}}$ were convoluted with the second-order wavelet functions $\psi_{\lambda_{2,j}}$ , and the second-order scattering propagators $U_{\lambda_{2,i,j}}$ were generated by the nonlinear modulus operation.

$$U_{\lambda_{2,i,j}}=\left|\left|X\left(t\right)*\psi_{\lambda_{1,i}}\right|*\psi_{\lambda_{2,j}}\right|,\;\;\;i=1\cdots n,j=1\cdots m.$$ (6)

5) The second-order wavelet scattering coefficients $S_{2,i,j}$ are obtained by the convolution of the second-order scattering propagators $U_{\lambda_{2,i,j}}$ and scaling function $\phi_{I}$ .

$$S_{2,i,j}X\left(t\right)=\left|\left|X\left(t\right)*\psi_{\lambda_{1,i}}\right|*\psi_{\lambda_{2,j}}\right|*\phi_{I},\;\;\;\;i=1\cdots n,j=1\cdots m.$$ (7)

**FIGURE 3:**
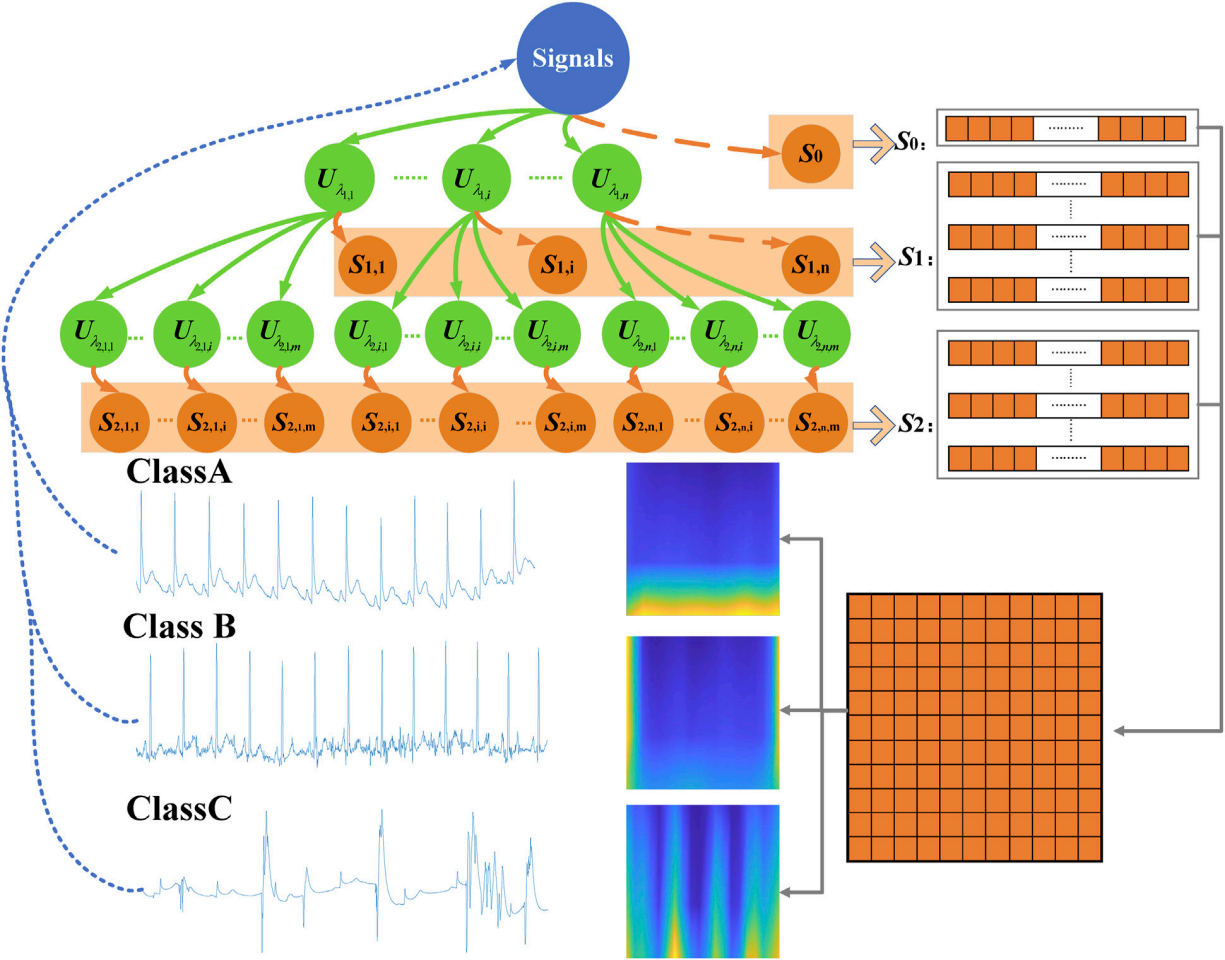
Three-layer wavelet scattering network.

The scattering network can contain more than three layers, but in practice, energy is dissipated with each iteration. Therefore, in this study, three layers were employed. The zero-order wavelet scattering coefficient $S_{0}$ mainly average the input ECG signal. The first-order wavelet scattering coefficient $S_{1}$ captures details lost in the first step, similar to the scale-invariant feature transformation function. The second-order wavelet scattering coefficient $S_{2}$ provides supplementary information that improves classification. The scattering characteristic matrix is composed of these three-layer scattering coefficients $S_{0},\;S_{1},\;S_{2}$ . Figure 3 displayed the three-layer wavelet scattering network, and three classes of signals.

**FIGURE 4:**
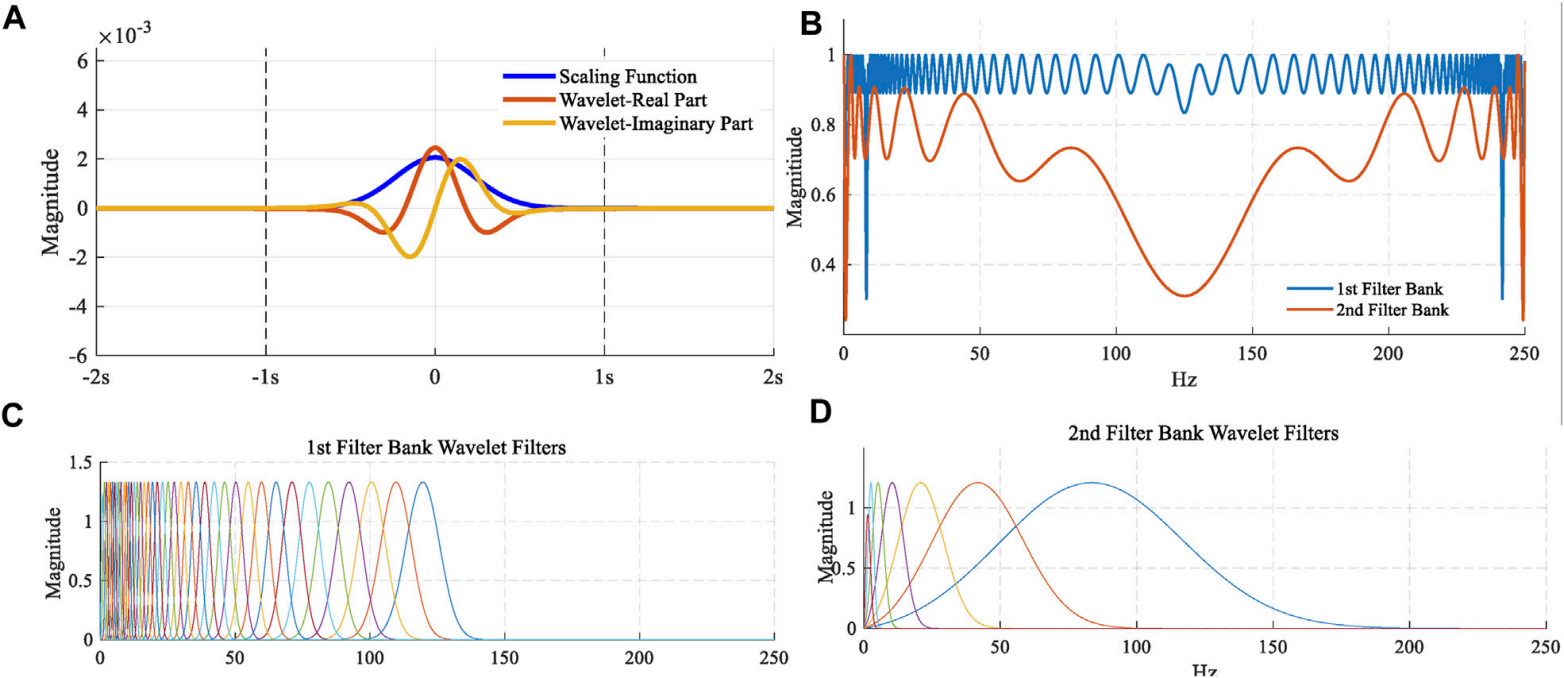
**(A)** Time-domain plots of the scale function and Morlet wavelet function; **(B)** Littlewood–Paley sums; **(C)** 41 first-order wavelet functions; **(D)** 7 second-order wavelet functions.

Figure 4**(A)** shows that time-domain plots of the scale function $\phi_{I}$ and Morlet wavelet function $\psi_{\lambda }$ were employed in the study. Invariance scale ***I*** in the scale function needs to be confirmed based on the length of data and sampling frequency. A total of 41 first-order wavelet functions and 7 second-order wavelet functions were used to build this wavelet scattering network, as shown in Figures 4 **(C)** and **(D)**. Also, Figure 4 **(B)** described the Littlewood–Paley sums for these scattering filter banks. Wavelet scattering networks could automatically extract feature extraction and could also reduce the signal dimension. The scattering characteristic matrix with dimension 81−by−20 was generated by this wavelet scattering network for one ECG signal with a length of 2,500 samples. For scattering coefficients of order 0, an input signal was first averaged using the scale function, which was the first matrix 1 × 20. For scattering coefficients of order 1, performing a continuous wavelet on the input signal yield a set of scalogram coefficients. Also, a modulus was applied to these coefficients and then the outputs were filtered with the wavelet low-pass filter yielding a set of order-1 scattering coefficients. It was the second matrix 41 × 20. For scattering coefficients of order 2, the same process was applied to the scalogram coefficients to obtain the third matrix 39 × 20. These three matrixes formed a scattering characteristic matrix with a dimension of 81 × 20. The columns (20) can be considered as the time dimension and 81 can be considered as the scale dimension. But this time dimension was after processed by average operation. Also, this scale dimension was also after processed by nonlinear modulus and averaging operators. It was different from the time-frequency map generated by the wavelet transform. A long short-term memory (LSTM) classifier with ADAM solver was used for classification.

## Evaluation Method

The evaluation indexes used in this study are sensitivity (***Se***), precision (***+P***), comprehensive index ***F***_**1**_ measure for A\B\C three quality grades, and modified accuracy (***mACC***). ***Se*** is the proportion of a certain class that has correctly predicted the total number of all real classes in the test dataset, including $Se_{A},Se_{B},Se_{C}$ . ***+P*** is the proportion of the certain class that has correctly predicted the total number of predicted to be this class in the test set, including $+P_{A},+P_{B},+P_{C}$ . ***F***_**1**_ measure includes $F_{1A},F_{1B},F_{1C}$ for class A/B/C, respectively. $mF_{1}$ is an average value of these three indexes.

$$F_{1A}=\frac{2\times TN_{A}}{N_{A}+T_{A}},\;F_{1B}=\frac{2\times TN_{B}}{N_{B}+T_{B}},\;F_{1C}=\frac{2\times TN_{C}}{N_{C}+T_{C}},$$ (8)

$$mF_{1}=\frac{1}{3}\left(F_{1A}+F_{1B}+F_{1C}\right),$$ (9)

$$\mathrm{mACC}=\frac{TN_{A}+TN_{B}+TN_{C}}{N\left(\mathrm{the}\;\mathrm{number}\;\mathrm{of}\;\mathrm{all}\;\mathrm{the}\;\mathrm{samples}\;\mathrm{in}\;\mathrm{the}\;\mathrm{test}\;\mathrm{set}\right)}.$$ (10)

where *TN*_A_, *TN*_B_, and *TN*_C_ are the number of signals accurately predicted as classes A, B, and C, respectively. *N*_A_, *N*_B_, and *N*_C_ are the number of all real class A, B, and C signals in the test set, respectively. *T*_A_, *T*_B_, and *T*_C_ are the number of all predicted to be class A, B, and C signals in the test set, respectively.

### Classification Model

The total number of signals in the database was 50,085. By pre-assessment, 1,029 lead-off and pure noise signals were eliminated. Remaining 49,056 signals were used to study the classification performance of the wavelet scattering network. In this study, a bi-directional long short-term memory (Bi-LSTM) network with the adaptive moment estimation (ADAM) solver was employed to train the classification model. The maximum number of epochs was 1,000. To reduce the amount of padding in the mini-batches, choose a mini-batch size of 490. The 10-fold cross-validation was employed to evaluate the classification performance of the model. All the segments were randomly divided into 10 groups. Also, the number of signals for each fold was 4,905.

### Real-Time Validation

**FIGURE 5:**
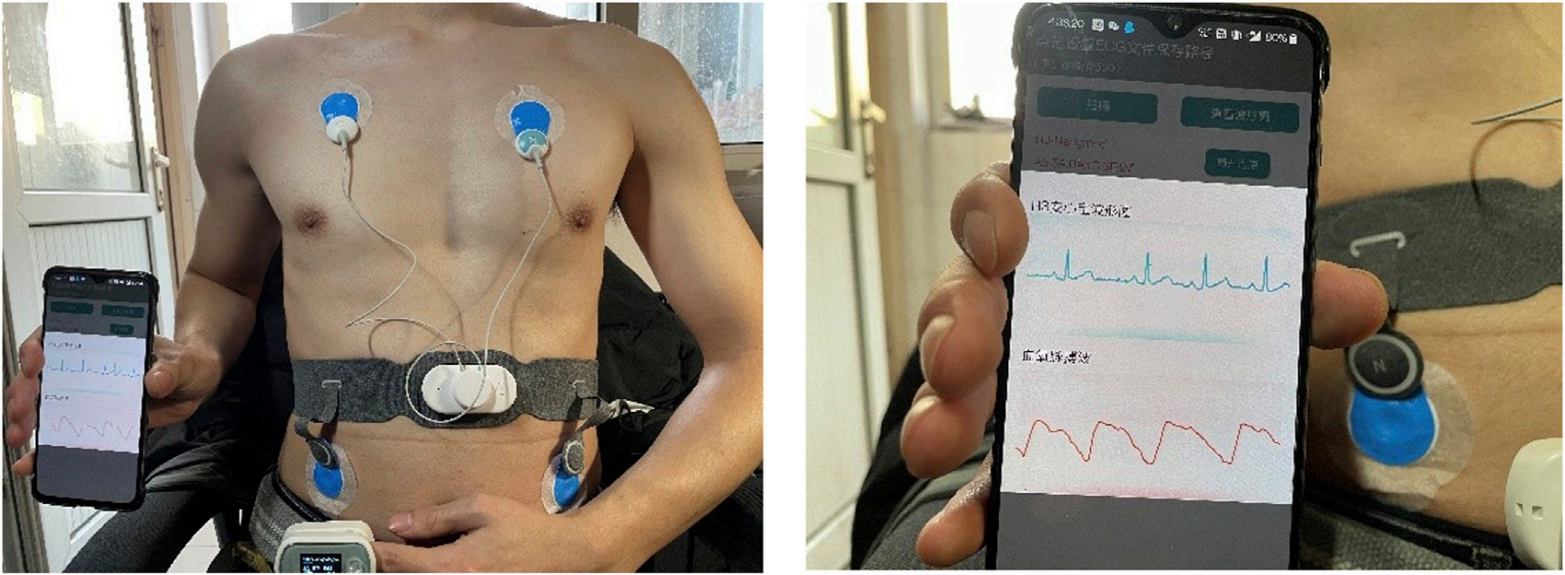
Schematic diagram of device wearing.

For real-time SQA performance analysis, the reliability of the whole wavelet scattering network method on real wearable dynamic ECG signals was tested. A Lenovo H3 dynamic ECG device was used for this experiment. Figure 5 shows the schematic diagram of device wearing. There were 60 subjects (24 females, 36 males) aged between 19 and 24 years in this experiment. For each subject, a 10-min duration of dynamic ECG signals was recorded under different physical activity conditions. The subjects wearing Lenovo H3 dynamic ECG devices were asked to perform different activities for 10-min duration, including sitting, walking, jogging, sitting, running, sitting, jumping, and sitting. In order to eliminate the interaction between two different physical activities, the subject was asked to sit and rest after strenuous exercise, such as running and jumping. The continuous wearable ECG signals collected by the Lenovo H3 device were transmitted to the phone via Bluetooth. The ECG signal was segmented with a frame length of 10 s and a hop-size of 1 s. The proposed wavelet scattering network SQA classifier evaluated the quality of the whole signal. The scattering characteristic matrix of the segment signals was generated by the proposed three-layer wavelet scattering network. A classification model was trained by all the signals (a total of 49,056) in the constructed database. By this classifier, the segmented signal was classified into different quality levels.

## Result

Tables 3 shows the confusion matrices of the classification results for an independent test set, and table 4 displays 11 evaluation indexes ( $\mathrm{mACC},mF_{1},Se_{A},Se_{B},Se_{C},+P_{A},+P_{B},+P_{C},F_{1A},F_{1B},F_{1C}$ ) of the average classification results for 10 cross-validation. As it turns out, the classification performance of the wavelet scattering network classifier was very great. The mean values of 11 evaluation indexes were nearly all greater than 98% (***mACC*** = 98.56%, ***mF***_**1**_ = 98.55%, ***Se***_A_ = 97.90%, ***Se***_B_ = 98.16%, ***Se***_C_ = 99.60%, + ***P***_A_ = 98.52%, + ***P***_B_ = 97.60%, + ***P***_C_ = 99.54%, ***F***_1A_ = 98.20%, ***F***_1B_ = 97.90%, ***F***_1C_ = 99.60%), only 97.90% for $Se_{A}\mathrm{and}\;F_{1B}$ , 97.6% for $+P_{B}$ . Particularly for class C, $Se_{C},+P_{C},F_{1C}$ were all greater than 99%.

**TABLE 3:** Confusion matrices of the classification results for an independent test set.

Confusion matrix		Pred			Sensitivity (Se)
		A	B	C	
Actual	A	1,578	37	1	97.65%
	B	23	1,597	8	98.10%
	C	2	8	1,645	99.40%
Precision (+P)		98.44%	97.26%	99.46%	98.39%

**TABLE 4:** Classified results of 27 SQI-based SVM classifiers and proposed method.

SQA method		*Se*_A_/%	*Se*_B_/%	*Se*_C_/%	*+P*_A_/%	*+P*_B_/%	*+P*_C_/%	*F*_1A_/%	*F*_1B_/%	*F*_1C_/%	*mF*_1_/%	*mAcc*/%
27 SQIs + SVM	10-fold cross-validation	87.79 ± 0.97	72.75 ± 1.47	96.06 ± 0.51	77.9 ± 1.09	83.17 ± 0.76	96.2 ± 0.46	82.52 ± 0.69	77.6 ± 0.69	96.1 ± 0.53	85.4 ± 0.44	85.33 ± 0.34
	Cross database validation, BUTQDB signals as training data, 2011 PhysioNet/CinC signals as testing data	61.06 ± 0.45	74.26 ± 0.61	90.74 ± 0.39	65.46 ± 0.87	68.47 ± 0.44	94.38 ± 0.56	63.18 ± 1.02	71.25 ± 0.98	92.53 ± 0.65	75.65 ± 0.33	75.06 ± 0.25
	Cross database validation, 2011 PhysioNet/CinC signals as training data, BUTQDB signals as testing data	63.48 ± 0.69	65.75 ± 0.94	98.03 ± 0.26	75.22 ± 0.47	62.06 ± 0.68	90.62 ± 0.67	68.85 ± 0.81	63.85 ± 0.78	94.18 ± 0.31	75.63 ± 0.84	77.65 ± 0.41
**Wavelet scattering + LSTM**	10-fold cross validation	**97.90** ± 0.54	**98.16** ± 0.58	**99.60** ± 0.39	**98.52** ± 0.81	**97.60** ± 0.94	**99.54** ± 0.16	**98.20** ± 0.85	**97.90** ± 0.84	**99.60** ± 0.29	**98.55** ± 0.40	**98.56** ± 0.39
	Cross database validation, BUTQDB signals as training data, 2011 PhysioNet/CinC signals as testing data	79.26 ± 1.50	83.32 ± 0.84	92.33 ± 0.67	80.38 ± 0.77	82.17 ± 1.39	92.82 ± 0.92	79.81 ± 1.01	82.74 ± 1.39	92.58 ± 0.87	85.04 ± 1.48	85.32 ± 0.99
	Cross database validation, 2011 PhysioNet/CinC signals as training data, BUTQDB signals as testing data	79.93 ± 1.03	75.68 ± 0.96	91.59 ± 0.84	84.44 ± 1.01	72.43 ± 0.99	90.11 ± 0.73	82.12 ± 0.88	74.02 ± 1.27	90.85 ± 0.65	82.32 ± 1.23	82.73 ± 1.11

The bold values were the results of the proposed method.

For classification performance comparisons, 27 typical SQA methods were selected (picaSQI (Li et al., 2014), tSQI (Liu F. et al., 2020), kSQI (Clifford et al., 2012), ELZ_compl_SQI (Zhang et al., 2014), sSQI (Clifford et al., 2012), bSQI_4 (Liu et al., 2018), DisEn_SQI (Li et al., 2015), pSQI (Li et al., 2008), bsSQI (Li et al., 2014), iSQI (Liu et al., 2019b), basSQI (Li et al., 2014), ApEn_SQI (Pincus et al., 1991), bSQI_2 (Li et al., 2008), HpSQI (Liu F. et al., 2020), FuzzyEn_SQI (Di Marco et al., 2012), SampEn_SQI (Chen et al., 2009), SDN_SQI (Everss-Villalba et al., 2017), eSQI (Li et al., 2014), MSQI (Tobon Vallejo et al., 2014), MpSQI, LpSQI (Liu F. et al., 2020), rsdSQI (Li et al., 2014), purSQI (Nemati et al., 2010), pcaSQI (Behar et al., 2013), PLI_SQI (Everss-Villalba et al., 2017), LZ_compl_SQI (Zhang et al., 2016), and hfSQI (Li et al., 2014)). These SQA methods were mainly based on the SQI indexes extracted from the time domain, frequency domain features, QRS waves, nonlinear characteristic, and others. The support vector machine (SVM) classifier was employed to train the classification model. As shown in Table 4, the classification accuracy of multi SQIs for 10-fold cross-validation was 85.33%.

Considering the overfitting influence of deep learning, cross-database validation was carried out to verify the generalization ability of this proposed method. All signals from BUTQDB were used as training data, while all signals from the 2011 PhysioNet/CinC were used as testing data, and vice versa, all signals from the 2011 PhysioNet/CinC were used as training data, while all signals from BUTQDB were used as testing data. Table 4 also displays the results of cross-database validation. For these two classifiers, classification accuracies were all greater than 80%. It was not as good as 10-fold cross-validation. The classification accuracy of multi SQIs for cross-database validation was about 75%.

## Discussion

In this study, we proposed a new SQA classification method based on a three-layer wavelet scattering network and built a wearable ECG quality database with 50,085 recordings for A/B/C three quality levels. The proposed SQA classifier had an excellent performance on this database ( $\mathrm{mACC}=98.56\%,mF_{1}=98.55\%$ ) for 10 cross-validation after all signals mixing. Particularly for class C signals, the proposed approach worked very well and the evaluation indexes were all greater than 99%. For class A and B signals, the results were slightly worse, but all greater than 97%. The wavelet scattering network used the deep convolution network architecture, but filter parameters were predefined. In this study, only the influence of the invariance scale was considered. Meanwhile, for performance comparisons, 27 typical SQA methods were selected to test the performance of this new database. Considering the overfitting influence of deep learning, cross-database validation and real-time validation were also carried out. The classification performance of cross-database validation was also admissible ( $\mathrm{mACC},mF_{1}$ ≥ 80%).

### Influence of Invariance Scale

In this study, the proposed three-layer wavelet scattering network was a deep learning framework which could extract complementary compact information automatically. The wavelet scattering network used the deep convolutional network architecture iterates over wavelet convolution, nonlinear modulus, and averaging (pooling) operators to compute higher-order scattering coefficients, which build translation invariant, stable, and informative signal representations. But the filters of the wavelet scattering network were predefined Morlet wavelets (Bruna and Mallat, 2013), which did not need to be learned from data. The Morlet wavelets were a localized waveform, having a better frequency resolution and stability to deformations, which could impose the separation of the different quality signals. The nonlinear modulus propagator recombines real and imaginary parts of complex wavelet coefficients, which could keep the low frequency averaging and obtain the translation invariant representation. Although the modulus operator removed the complex phase and lost information about the high frequencies, it kept the temporal variation of the multiscale envelopes. Also, the high frequencies information lost by the pooling can be recovered as wavelet coefficients in the next layers as the wavelet transform was a redundant representation. High order scattering coefficients could characterize transient phenomena of the different noises from free living. To recover this high-frequency information, a new wavelet transform was implemented to the signal in the next layers before the nonlinear modulus and pooling were performed.

**FIGURE 6:**
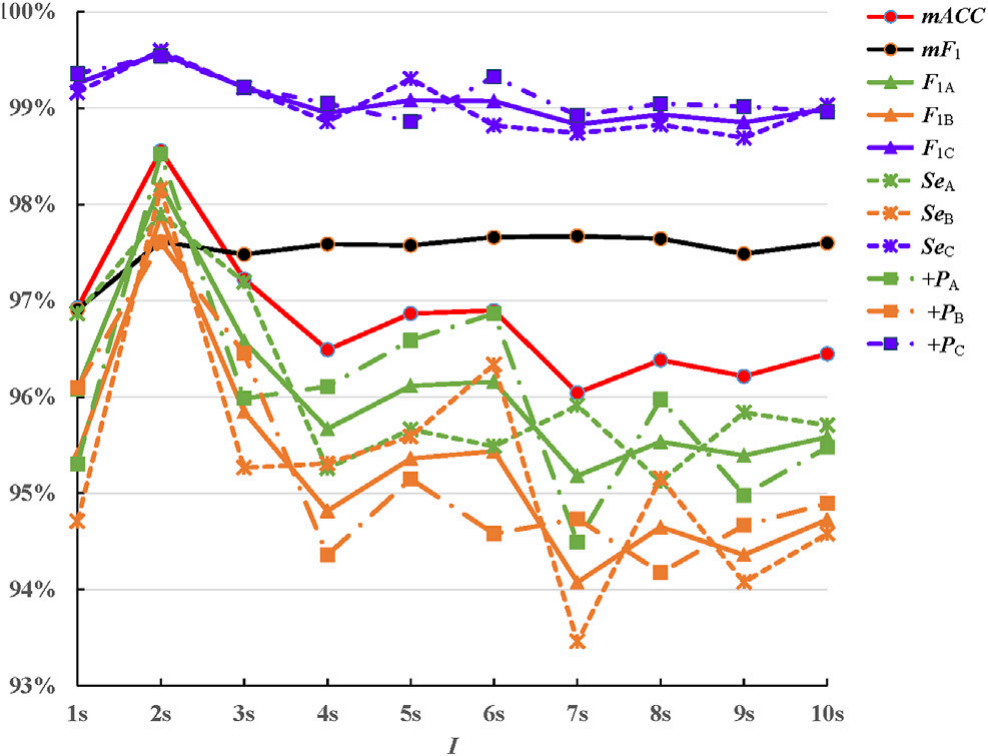
Classification results for invariance scale I=1s,2s,⋯8s,9s,10s .

The invariance scale was also termed as the interval of time-shift invariance, which was defined by the size of the time averaging window. The influence of this parameter was also considered in this study. Also, the scattering coefficients were computed at scales I=1s,2s,⋯8s,9s,10s. Figure 6 displayed the classification results. As shown in Figure 6, the invariance scale ***I*** changing had less influence on the accuracy. All 11 evaluating indexes were above 93%. But obviously, when the invariance scale was set to be 2s, the classification performance was best. The scale ***I*** controlled the amount of translation invariance. When it was too small, noises produced by gross movements, such as severe drifting baselines, would miss some. When it was too large, the convolution would lose partly high frequency information. In this study, 2s was the best choice for the invariance scale. The classification performances showed small differences in the changing of the invariance scale. The variability within each class A/B/C was not due to translation, but due to time-domain deformations and spectrum noise.

### Comparing With Other SQA Methods

**FIGURE 7:**
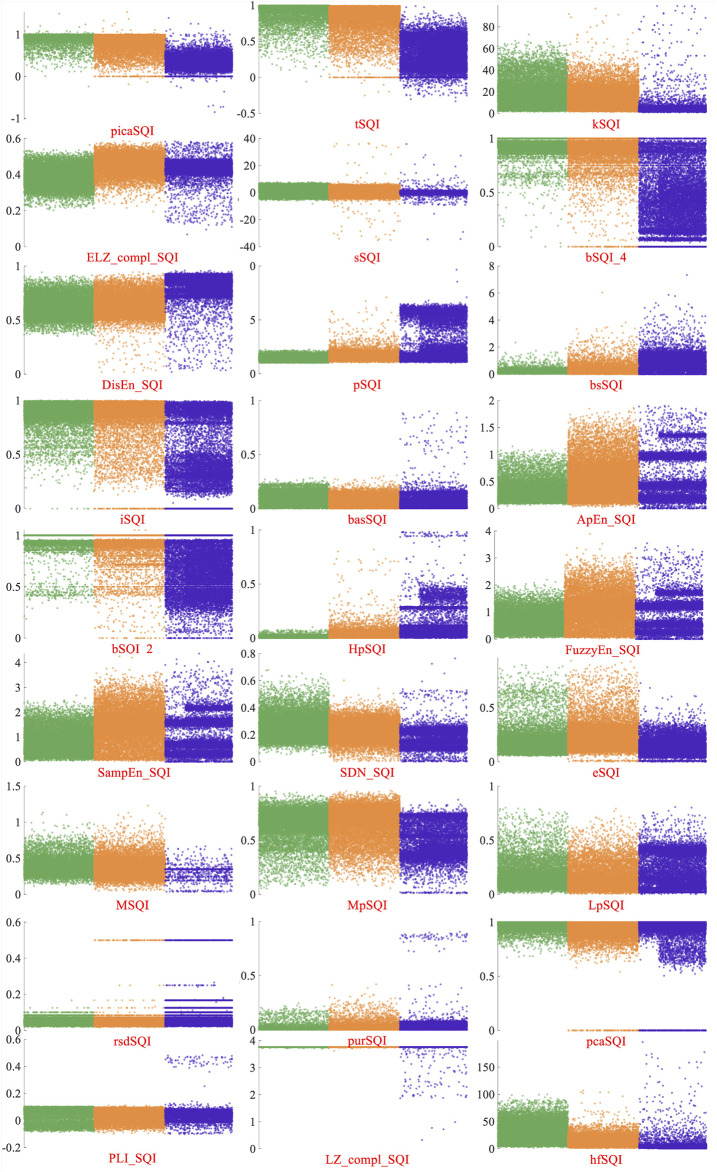
Distribution of 27 SQIs on the A/B/C quality levels signals. Green, orange, and blue dots represent class A/B/C signals, respectively.

At present, most studies about the signal-quality assessment divided the ECG signals into acceptable and unacceptable. There are fewer public databases with three quality levels. For performance comparisons, 27 typical SQA methods were selected to test the performance of this new database. These methods mostly had better performance on the database including two classes of ECG signals. Multiple SQI feature–based classifiers were lower than the proposed novel classification method. In order to analyze the performances of these SQIs better, Figure 7 displays the distribution of these SQIs on the A/B/C quality levels signals. Green, orange, and blue dots represent class A/B/C signals, respectively. The SQIs, which are only based on the QRS waves, such as bSQI-4 and bSQI-2, were defined by the comparison of four or two QRS wave detectors on a single-lead signal. They had good performance on the database with two classes of ECG signals ( mACC>93% ) (Liu et al., 2018), but class A signals are mixed up with class B signals, as shown in Figure 7. It is because that QRS wave of class B signals also could be detected accurately. The performance of tSQI and picaSQI was slightly better. These two SQIs were computed not only based on the QRS wave but also based on morphology consistency and nonlinear characteristic (Li et al., 2014; Liu F. et al., 2020). The tSQI was defined as the morphology consistency of any two ECG beats within a fixed time window (Li et al., 2014), and the picaSQI was defined as a periodicity measure of the ECG waveform nonlinear characteristic (Liu F. et al., 2020). For other SQIs based on time and frequency domain features, the distribution ranges for A/B/C quality level signals had a large overlap region. The class B signals were mostly contaminated by the noise with high frequency and low amplitude, which can make partly class B signals detected to be class A or C.

It should be noted that all these 27 SQIs that we selected were unlikely to be the optimal indexes. We tried to pick as many quality metrics as possible, but it is impossible to pick all of them. Meanwhile, because some SQIs were published in a theoretical way without the executable program, and some literature works lacked detailed necessary preprocessing operations, some SQIs were coded by us. Thus, the classing results in this study could be different from those in the other studies, but the differences are unimportant.

In this study, all the programs were implemented using MATLAB 2020a. Table 5 illustrates the mean time costs and standard deviation values of the 12 SQIs and the proposed method by analyzing 49,056 10-s ECG segments in the database. As shown in Table 5, the proposed method was the most time-efficient compared with 12 SQIs. Also, 18.25 ms was not a long-time cost for 10-s ECG segments.

**TABLE 5:** Mean time costs and standard deviation values of 12 SQIs and proposed method.

Method	Mean time/ms	SD/ms
picaSQI (Li et al., 2014)	4.04	0.41
tSQI (Liu et al., 2020a)	1.20	0.20
kSQI (Clifford et al., 2012)	1.14	0.13
ELZ_compl_SQI (Zhang et al., 2014)	31.74	6.55
sSQI (Clifford et al., 2012)	1.05	0.23
bSQI_4 (Liu et al., 2018)	6.83	0.65
DisEn_SQI (Li et al., 2015)	25.28	8.31
pSQI (Li et al., 2008)	2.15	0.25
bsSQI (Li et al., 2014)	2.01	0.30
iSQI (Liu et al., 2019b)	1.08	0.26
basSQI (Li et al., 2014)	3.31	0.43
ApEn_SQI (Pincus et al., 1991)	30.41	3.60
**Proposed method**	**18.25**	**1.38**

The bold values were the time cost and standard deviation of the proposed method.

### Real-Time Validation and Cross-Database Validation Analysis

**FIGURE 8:**
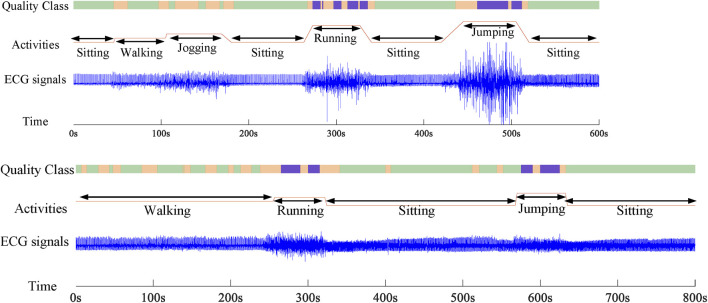
Two records about 10-min duration of dynamical ECG signals, physical activities, and evaluation results.

For real-time SQA performance analysis, the reliability of the whole wavelet scattering networks method on real wearable ECG signals was tested. Figure 8 displays two segments of 10-min duration of dynamical ECG signals, physical activities, and evaluation results. As it turns out, under the sitting and walking conditions, the quality of the ECG signal was very good and all signals are assessed as class A, which can be used for the cardiovascular disease diagnosis. In the jogging condition, some signals were contaminated by weak artifacts and assessed as class B. But they could not affect the R wave identification, which can be used for the heart rate measure. In the running and jumping conditions, most of the signals were contaminated by seriously large noises caused by violent motion and assessed as class C. These signals will interfere with the diagnosis of the cardiovascular disease and need to be removed. Also, the proposed SQA method could identify the changing of the position. During the changing stage, there were some fluctuations in the signal. These signals were assessed as class B. The evaluation results show that the proposed wavelet scattering network SQA classifier framework has capability to assess wearable dynamic ECG signal quality.

In this study, cross-database testing was also carried out to verify the generalization ability of this proposed method. As the results show, classification accuracy was greater than 80%. The performance of cross-database validation was not good as 10-fold cross-validation. But for class C, $Se_{C},+P_{C},F_{1C}$ were all greater than 90%. The reason for this phenomenon is the great difference between these two databases. The signals from BUTQDB were single-lead ECGs with three quality classes, while the signals from the 2011 PhysioNet/CinC Challenge were 12-lead ECGs with two quality classes. Although we, based on the scores in Liu et al. (2018), annotated all the leads individually, there are still differences in the re-annotating. The morphological characteristics of class C signals are obvious, and the evaluation of experts is relatively consistent. However, the difference in morphological characteristics between class A and class B signals is not particularly obvious, the evaluations between different experts are different. If all the signals from the 2011 PhysioNet/CinC Challenge were re-annotated based on the criteria of BUTQDB strictly, the performance of cross-database validation will be better. However, it will need more time cost.

In this study, the approach adding noise to clean data was used to balance the database classes. Also, those clear recordings from classes A and B used to upsample class C, were put back into the original classes. It needs to be considered if this synthetic noise is going to play a role in the classification results. If one clear recording from class A is in the training set and it was also corrupted by noises used to upsample the class C in the test set. If this recording includes some information on the test set, this information will tend to judge this recording as class A. However, it was a contaminated recording and labeled as class C. Therefore, if there is some information generated by balancing the dataset on the test set, classification accuracy will be reduced. However, the classification performance of this method was very good. Therefore, the approach used to balance the dataset did not influence the classification results. The testing of real signals without synthetic addition was also carried out to consider the influence of this balancing data approach. A new database was built with 5,687 recordings without any synthetic noise added, class A: 2,000, class B: 2,000, and class C: 1,687. We used 30% of the data for testing and the remaining 70% of the data for training. The values of 11 evaluation indexes were nearly all greater than 90%, ***mACC*** = 94.05%, ***mF***_**1**_ = 94.03%, ***Se***_A_ = 93.35%, ***Se***_B_ = 91.25%, ***Se***_C_ = 97.55%, + ***P***_A_ = 94.44%, + ***P***_B_ = 92.73%, + ***P***_C_ = 94.94%, ***F***_1A_ = 93.89%, ***F***_1B_ = 91.98%, and ***F***_1C_ = 96.23%*.* The reduction in the data volume reduced the accuracy of the model, which was also acceptable. The approach of adding noise to clean data to balance the database classes was also used in Clifford et al. (2012).

Most notably, there is no unified evaluation criterion to determine the quality levels of wearable ECG at present. Different databases provide different evaluation methods. For example, the data in the 2011 PhysioNet/CinC Challenge are 12-lead recordings, having a length of 10 s 3–18 annotators marking each signal, and each record was assigned to one of the three groups (acceptable 773, indeterminate 2, and unacceptable 225) based on the average score. Some studies considered that the label of “acceptable” or “unacceptable” was for the whole 12 channels, not for the single channel. Therefore, they re-labeled each channel (Clifford et al., 2012; Liu et al., 2018), and balanced the classes by adding noise to some of the clean data. However, in the BUTQDB database, 18 single-lead signals longer than 24 h were recorded using the Bittium Faros 180 device. The parts of signals were selected to be grouped into three quality levels based on the labels annotated by three experts. Also, some studies constructed a manually annotated gold standard, collected and annotated ECG recordings by themselves (Redmond et al., 2012; Satija et al., 2017; Liu et al., 2019b; Smital et al., 2020). Different classification standards and annotating methods could have great influence on the SQA performance.

### Limitations and Prospects

Wearable electrocardiogram quality assessment is quite crucial for cardiovascular disease prevention and diagnosis. It is also an important issue for wearable device development. Although the proposed new method had great performance on the quality assessment, it was not very well for cross-database validation. The main reason is the difference between annotation methods and classification grades. For future work, uniform and standardized evaluation criterion is quite crucial for the wearable ECG quality assessment.

## Conclusion

This study aimed to provide a method to classify wearable dynamic ECG signals into three grades: high quality (A), medium quality (B), and low quality (C). A new SQA classification method based on a three-layer wavelet scattering network and transfer learning LSTM was proposed, and a wearable ECG quality database with 50,085 recordings for three quality grades was built. In order to avoid the adverse impact of invalid samples on the training models, the quality pre-assessment was used to delete the lead-fall signals and pure noise. A three-layer wavelet scattering network was performed on the selected 10-s-long signal segments, which can extract more systematic and comprehensive characteristics by analyzing the signals thoroughly and deeply. The Bi-LSTM network with ADAM solver was employed to train the classification model. The 11 evaluating indexes ( $\mathrm{mACC},mF_{1},Se_{A},Se_{B},Se_{C},+P_{A},+P_{B},+P_{C},F_{1A},F_{1B},F_{1C}$ ) were 98.56%, 98.55%, 98.52%, 97.60%, 99.54%, 98.20%, 97.90%, 99.60%, 97.90%, 98.16%, and 99.60%, respectively, suggesting that the proposed method can effectively separate three quality grades of wearable ECG signals. For efficacy validation, this method was applied on the real-world data collected using the Lenovo H3 dynamic ECG device. This method had the ability to detect noise signals produced by vigorous activities. With the high computational efficiency, it will have a good application on wearable ECG devices, including removing contaminating signals and selecting high-quality signal segments for CVD diagnosis and analysis. This study verified the feasibility of applying the wavelet scattering network model to wearable ECG signal-quality assessment. Also, the general framework of this classification method proposed in this study was sufficiently flexible to be used in any given situation.

## Data Availability

The raw data supporting the conclusion of this article will be made available by the authors, without undue reservation.
